# Cardiovascular and mortality benefits of sodium–glucose co-transporter-2 inhibitors in patients with type 2 diabetes mellitus: CVD-Real Catalonia

**DOI:** 10.1186/s12933-021-01323-5

**Published:** 2021-07-09

**Authors:** Jordi Real, Bogdan Vlacho, Emilio Ortega, Joan Antoni Vallés, Manel Mata-Cases, Esmeralda Castelblanco, Eric T. Wittbrodt, Peter Fenici, Mikhail Kosiborod, Dídac Mauricio, Josep Franch-Nadal

**Affiliations:** 1 DAP‑Cat Group, Unitat de Suport a la Recerca Barcelona, Fundació Institut Universitari per a la recerca a l’Atenció Primària de Salut Jordi Gol i Gurina (IDIAPJGol), Barcelona, Spain; 2 CIBER of Diabetes and Associated Metabolic Diseases (CIBERDEM), Instituto de Salud Carlos III (ISCIII), Madrid, Spain; 3 Department of Endocrinology and Nutrition, Institut d’Investigacions Biomèdiques August Pi i Suñer, Hospital Clinic, Barcelona, Spain; 4 CIBER of Physiopathology of Obesity and Nutrition (CIBEROBN), Instituto de Salud Carlos III (ISCIII), Madrid, Spain; 5 Drug Area, Gerència d’Atenció Primaria, Institut Català de la Salut, Barcelona, Spain; 6 Primary Health Care Center La Mina, Gerència d’Àmbit d’Atenció Primària Barcelona Ciutat, Institut Català de la Salut, Sant Adrià de Besòs, Spain; 7 AstraZeneca, Gaithersburg, MD USA; 8 Cardiovascular Renal Metabolisms, BioPharmaceuticals Global Medical, AstraZeneca, Cambridge, UK; 9 Saint Luke’s Mid America Heart Institute and University of Missouri-Kansas City, Kansas City, MO USA; 10 Department of Endocrinology and Nutrition, Hospital Universitari de la Santa Creu i Sant Pau, Autonomous Universtity of Barcelona, Sant Quintí, 89, 08041 Barcelona, Spain; 11 Departament of Medicine, University of Vic-Central University of Catalonia, Vic, Barcelona, Spain; 12 Primary Health Care Center Raval Sud, Gerència d’Atenció Primaria, Institut Català de la Salut, Av. Drassanes, 17-21, 08001 Barcelona, Spain

**Keywords:** SGLT2i, Heart failure, All-cause mortality, Type 2 diabetes mellitus

## Abstract

**Background:**

Evidence from prospective cardiovascular (CV) outcome trials in type 2 diabetes (T2DM) patients supports the use of sodium–glucose co-transporter-2 inhibitors (SGLT2i) to reduce the risk of CV events. In this study, we compared the risk of several CV outcomes between new users of SGLT2i and other glucose-lowering drugs (oGLDs) in Catalonia, Spain.

**Methods:**

CVD-REAL Catalonia was a retrospective cohort study using real-world data routinely collected between 2013 and 2016. The cohorts of new users of SGLT2i and oGLDs were matched by propensity score on a 1:1 ratio. We compared the incidence rates and hazard ratio (HR) for all-cause death, hospitalization for heart failure, chronic kidney disease, and modified major adverse CV event (MACE; all-cause mortality, myocardial infarction, or stroke).

**Results:**

After propensity score matching, 12,917 new users were included in each group. About 27% of users had a previous history of CV disease. In the SGLT2i group, the exposure time was 60% for dapagliflozin, 26% for empagliflozin and 14% for canagliflozin. The use of SGLT2i was associated with a lower risk of heart failure (HR: 0.59; 95% confidence interval [CI] 0.47–0.74; p < 0.001), all-cause death (HR = 0.41; 95% CI 0.31–0.54; p < 0.001), all-cause death or heart failure (HR = 0.55; 95% CI 0.47–0.63; p < 0.001), modified MACE (HR = 0.62; 95% CI 0.52–0.74; p < 0.001), and chronic kidney disease (HR = 0.66; 95% CI 0.54–0.80; p < 0.001).

**Conclusions:**

In this large, retrospective observational study of patients with T2DM from a Catalonia, initiation of SGLT-2i was associated with lower risk of mortality, as well as heart failure and CKD.

**Supplementary Information:**

The online version contains supplementary material available at 10.1186/s12933-021-01323-5.

## Introduction

Cardiovascular disease (CVD) is the leading cause of morbidity and mortality among individuals with type 2 diabetes mellitus (T2DM) [1, 2]. Indeed, people with T2DM have a two- to fourfold increased risk for coronary heart disease compared to subjects without diabetes [3, 4], and heart failure (HF) is another common complication of T2DM associated with high risk of CV death [5]. Moreover, different studies have shown that glycaemic control does not significantly reduce CV risk, stressing the need for novel treatments that can prevent the development of CVD complications independent of glucose lowering [6–8].

So far, meta-analyses of prospective CV outcome trials (CVOTs) have consistently shown that treatment with sodium–glucose co-transporter-2 inhibitors (SGLT2i) modestly reduces the incidence of major adverse cardiovascular outcomes (MACE) and have a consistent and robust beneficial effect on hospitalizations for heart failure and progression of kidney disease regardless of prior history of CVD or established CKD [9–12]. Besides, trials studying kidney function as a secondary outcome or subgroup analysis have reported additional positive benefits, including reduced risk of end-stage renal disease and renal failure death [13–17].

In the real-world setting, several large multinational, observational studies assessed the comparative effectiveness of initiating treatment with a SGLT2i vs. other glucose-lowering drugs (oGLDs) (CVD-REAL; NCT02993614). The results of the CVD-REAL 1 and 2 studies showed that the use of SGLT2i was associated with decreased risk of hospitalisation for HF and all-cause mortality in subjects with a broad range of CV risk [18, 19]. Moreover, the CVD-REAL Nordic (conducted in Denmark, Norway, and Sweden) reported an association between SGLT2i initiation and a decreased risk of CV mortality and MACE compared with oGLDs [20]. Regarding dapagliflozin in particular, treatment initiation was associated with a lower risk of nonfatal myocardial infarction (MI), nonfatal stroke or CV death, and all-cause mortality compared with dipeptidyl peptidase-4 inhibitors (DPP-4i) [21]. The results from the CVD-REAL 2 Study (conducted in Asia Pacific, the Middle East, North America and also Spain) confirmed that the use of SGLT2i was associated with a lower risk of death, death or hospitalisation for HF, MI, and stroke in a broad range of T2DM subjects with or without CVD [19]. Lastly, the recent CVD-REAL 3 Study (conducted in Israel, Italy, Japan, Taiwan, and the UK) showed that new users of an SGLT2i had a slower rate of kidney function decline and a lower risk of clinically meaningful kidney events compared with those on oGLDs [22]. Other retrospective observational cohort studies conducted by independent groups in different populations and applying different methodologies have produced consistent results to what already observed in the CVD REAL studies [23–26].

Available observational, real-world studies suggested that there might be a class effect of SGLT2i on CV and renal outcomes, and that the benefits could be extended to a broader population with diabetes (i.e., with a broad CV risk profile and different stages of renal disease) [27, 28]. As such, SGLT2i are nowadays endorsed as second line therapy by European and American clinical guidelines for patients with T2DM and CV risk factors, history of HF, or chronic kidney disease (CKD) [29, 30]. However, it is important to examine whether these results can be replicated in Southern Europe areas other than Italy, the only countries of this region included and only regarding the assessment of renal outcomes in the CVD REAL available studies [19]. It is also important to consider that Fadini et al. reported that there are country-specific differences in the profile and characteristics of patients initiating dapagliflozin in Southern Europe [31]. In their real-world practice study, including patients from Italy, Spain, and Greece, the authors found that the SGLT2i dapagliflozin was initiated at different stages depending on the country, with significant differences regarding age, mean T2DM duration, and the presence of comorbidities (e.g., retinopathy, prior stroke, MI, or CVD) [31]. The authors concluded that the observed geographic heterogeneity might not only have an impact in the glucose-lowering effectiveness of SGLT2i but also in the protective CV and renal outcomes. Moreover, the CV risk factors and the prevalence of CVD in persons with T2DM differ across regions in Europe [32, 33]. For example, Spain has a lower prevalence of CVD than Northern European countries but higher than Italy [33]. Considering these potential differences, we hypothesised that the CVD-Real outcomes could be different when the general T2D population is estimated in a Southern European country—region such as Catalonia (Spain).

Based on the need to confirm the applicability of results from other observational, real-world studies, the primary aim of the present study was to investigate, in a primary care population database in Catalonia (Spain), whether the risk for HF among users initiating SGLT2i was different from those initiating oGLDs. Secondarily, we aimed to compare all-cause mortality, modified MACE (i.e., all-cause mortality, MI, or stroke), all-cause mortality or HF, MI, stroke, and CKD between these two groups of new users.

## Methods

### Study design and data sources

This was a retrospective cohort study of adult subjects with T2DM between January 2013 and December 2016. Data from patients were extracted from the electronic medical records of the SIDIAP database (Information System for the development of Primary Care Research) and the CMDB register (Hospital Discharge Records in the National Health System) from the Catalan Institute of Health (ICS) [34]. The assigned population of the ICS is around 5.6 million individuals (approximately 74% of the total Catalan population) organised in 288 primary care teams. These electronic datasets include anonymised demographic data, visits to primary care services, specialist referrals, diagnoses and procedure codes (International Classification of Diseases [ICD]-9 and 10 systems), clinical data, laboratory test results, all-cause mortality, information on drugs prescriptions, and pharmacy dispensations.

### Subjects

To identify patients initiating glucose-lowering treatment with either an SGLT2i (canagliflozin, dapagliflozin, or empagliflozin) or oGLDs in our databases we used the codes of the Anatomical Therapeutic Chemical (ACT) classification system from the World Health Organization (WHO) [35]. New users were defined as individuals with a registered prescription or drug dispensation (either as initial or add-on therapy) for any SGLT-2i (i.e., canagliflozin, dapagliflozin or empagliflozin) or oGLDs, including fixed-dose combinations, with no prior prescriptions of an SGLT-2i during the preceding year [18]. If the subject in the oGLDs group started with more than one drug, one of these drugs was randomly selected as the index drug.

New users were eligible if they initiated treatment with either an SGLT2i or oGLD between the 1st December 2013 and the 31st December 2016. In our approach, all episodes of SGLT-2i and oGLD initiation were eligible to be included, thus one patient might be included more than once and might have contributed with more than one episode of new glucose-lowering medication initiation for different drug classes (e.g., SGLT-2i and various classes of oGLDs) and at different time points. Furthermore, subjects were not selected hierarchically; therefore, the potential risk period from the oGLD group was removed, which could lead to mortality bias [36]. This methodological approach allowed all oGLD exposure episodes to contribute to oGLD estimates and all SGLT2i exposure episodes to contribute to SGLT2i estimates. Additionally, the groups were built with 1:1 propensity matching for new glucose-lowering agent (oGLD or SGLT2i) episodes rather than individual subjects, taking into account each drug episode within subjects. This methodologic approach was made in order to minimize immortal time bias. Moreover, subjects were included in the study by the index date (defined as the prescription/dispensation date for the new SGLT2i or oGLD) were ≥ 18 years and had more than 1-year medical history in the database. Subjects with a diagnosis of type 1 diabetes (ICD10 code E10), gestational diabetes (ICD10 code O24.4), or on dialysis treatment (ICD10 code Z49) were excluded from the study. All subjects were followed from the index date to the earliest end of the use of the given treatment or the date that the subject moved to another healthcare region not served by ICS (thus withdrawn from the database), last date of data collection, or to the death date in the on treatment approach. In the intention to treat approach, subjects were followed until the last date of data collection independently if they discontinued their index treatment or switched to another treatment.

### Variables

Variables were captured for all patients at the index date inclusive or before (1 year prior to the index date). At baseline, we collected data for social-demographic characteristics, including age, gender, toxic habits (smoking), and deprivation index (DI). The DI assesses five socioeconomic indicators related with work and education (i.e., unemployment, manual and eventual workers, and insufficient education overall and in young people) extracted from census tracks [37]. It detects small areas of large cities in Spain with unfavorable socioeconomic characteristics and is associated with overall mortality. The higher is the DI, the worse the social deprivation is.

Clinical characteristics included diabetes-related parameters [e.g., glicated haemoglobin (HbA1c) and diabetes duration], concomitant medications, comorbidities, and laboratory parameters (with the baseline value defined as the last available value during the prior year including the index date). During the follow-up, we collected data (ICD10 diagnostic codes) on the following events as single outcomes: all-cause mortality, HF [hospitalisation for HF or diagnostic code (ICD10: I50), atrial fibrillation (ICD10: I48), stroke (ICD10: I60–I62), ischemic stroke (ICD10: I63–I64), MI (ICD10: I21–I22), and CKD (ICD10: N18, N08.3, E11.2]. In addition, we also analysed the following composite outcomes: (a) all-cause mortality or HF (hospitalisation for HF or diagnostic code), and (b) modified MACE, defined as all-cause mortality, MI or stroke.

### Statistical methods

The SGLT-2i group was matched to the oGLDs group (comparator) based on propensity score and calendar period of study entry. For matching, we used the nearest neighbour calliper width of 0.25 multiplied by the standard deviation (SD) of the propensity score distribution [38]. The variables considered for the estimation of the propensity score included age at study date entry (index date), gender, CV risk factors, indicators of diabetes severity, and use of concomitant medications (Additional file 1: Table S1). Once the propensity score was performed, new treatment episodes of initiators of SGLT2i and oGLDs were matched 1:1.

The baseline characteristics for each cohort were summarised by frequencies and percentages for categorial variable, and as mean (± SD or median and quartiles) for continuous and count variables. For the main on treatment analysis, we calculated the person-time at risk for each patient as the length of the index exposure episode, defined as the number of days from the day after the index prescription start date to the last day of follow-up. For each outcome of interest, the crude incidence rate (IR) in each index exposure group was estimated as the number of incident events divided by the total number of patient-years (PY) at risk and was expressed as per 100 PY. The IRs for the SGLT2i group and oGLD group were then compared using a hazard ratio (HR) and the corresponding 95% confidence interval (CI). This analysis was performed using Cox proportional hazards regression by clusters (patient ID) with robust estimation of standard errors. Both crude and adjusted HR were estimated for all endpoints. As covariates for adjustment, we used gender, age, T2DM duration, hypertension, body mass index (BMI), use of antihypertensive drugs, and HF or MI (Additional file 1: Table S2). In addition, Kaplan–Meier plots were are also generated for each of the analysed outcomes. A sensitivity analysis was further conducted with an intent-to-treat approach with those subjects who continued in the study even after they discontinued their index treatment or switched to another treatment. Finally, a subgroup analysis was performed within the following pre-specified subgroups: prior CV disease, prior heart failure, ≥ 65 years of age at the index date, gender, prior kidney disease, and baseline treatment with antihypertensive drugs, insulin, sulfonylureas, GLP1-RA, thiazolidinedione and statins. All statistical analyses were performed using the free R statistical software, version 3.6.1 (https://www.r-project.org/). The source code is available at https://github.com/jrealgatius/CVD_REAL_OP2, and a dashboard with interactive Additional material at https://dapcat.shinyapps.io/CVD_REAL.

## Results

A total of 239,733 subjects with T2DM were identified as new user episodes of glucose-lowering drugs during the observational period in the SIDIAP database, with 226,452 of them (94.5%) initiating treatment with and the remaining 13,281 with an SGLT2i (5.5%). Baseline characteristics before propensity-score matching are shown in Additional file 1: Table S3. Briefly, we observed that SGLT2i new users were younger, with a longer duration of T2DM, higher BMI, and poorer glycemic control than oGLDs new users. Moreover, the proportion of patients with microvascular disease, hypertension, previous MI, and peripheral artery disease (PAD) was higher among those initiating an SGLT2i.

After propensity-score matching, we obtained well-balanced cohorts for patients' baseline characteristics with standardised differences for all of the variables less than 10%, which resulted in a total of 25,834 new user episodes, 12,917 in each drug cohort (Additional file 1: Figures S1 and S2; http://dapcat.shinyapps.io/CVD_REAL). In the overall population, more than two-thirds of users at the moment of initiation with a new glucose-lowering drug had a diabetes duration of ≥ 5 years (77.7%), suboptimal glycemic control (mean HbA1c = 8.69%; SD = 4.8), and nearly two-thirds (65.1%) were on statins treatment (Table 1).

**Table 1:** Baseline characteristics of the study participants after propensity score matching

	Total population N = 25,834	oGLD N = 12,917	SGLT2i N = 12,917	Standardized mean differences
Age, mean (SD), years	62.9 (11.1)	62.8 (11.8)	62.9 (10.4)	0.007
Gender, n (%), females	11,331 (43.9)	5682 (44.0)	5649 (43.7)	0.005
Smoking, n (%)	4145 (16.3)	2081 (16.3)	2064 (16.2)	0.004
Diabetes duration ≥ 5 years, n (%)	20,065 (77.7)	10,037 (77.7)	10,028 (77.6)	0.002
HbA1c (%), mean (SD)	8.69 (1.57)	8.79 (1.64)	8.59 (1.48)	0.128
Missing’s, n (%)	5980 (23.1)	2948 (22.8)	3032 (23.5)	
Deprivation index (medium–high), n (%)	12,356 (47.9)	6156 (47.6)	6200 (48.0)	0.016
Comorbidities, n (%)				
Cardiovascular disease	7019 (27.2)	3466 (26.8)	3553 (27.5)	0.015
Heart failure	1421 (5.5)	692 (5.4)	729 (5.6)	0.013
Myocardial infarction	1624 (6.3)	800 (6.2)	824 (6.4)	0.008
Unstable angina	383 (1.5)	197 (1.5)	186 (1.4)	0.007
Atrial fibrillation	1343 (5.2)	668 (5.2)	675 (5.2)	0.002
Stroke	1488 (5.8)	752 (5.8)	736 (5.7)	0.005
Chronic kidney disease	1697 (6.6)	1021 (7.9)	676 (5.2)	0.108
Peripheral artery disease	1895 (7.3)	942 (7.3)	953 (7.4)	0.003
Microvascular disease^a^	5749 (22.3)	2884 (22.3)	2865 (22.2)	0.004
Cancer	2975 (11.5)	1589 (12.3)	1386 (10.7)	0.049
eGFR, mL/min/1.73 m^2^, mean (SD)	58.8 (4.82)	58.5 (5.5)	59.0 (3.98)	0.100
Missing’s, n (%)	6058 (23.4)	3003 (23.2)	3055 (23.7)	
Concomitant medications, n (%)				
*Anti-hypertensives*	19,453 (75.3)	9645 (74.7)	9808 (75.9)	0.029
ACE inhibitors	9891 (38.3)	4950 (38.3)	4941 (38.3)	0.001
Angiotensin II receptor blockers	8577 (33.2)	4272 (33.1)	4305 (33.3)	0.005
Beta blockers	6926 (26.8)	3441 (26.6)	3485 (27.0)	0.008
Calcium channel blockers	540 (2.1)	290 (2.3)	250 (1.9)	0.022
Thiazides	2431 (9.4)	1219 (9.4)	1212 (9.4)	0.002
Loop-diuretics	3008 (11.6)	1492 (11.6)	1516 (11.7)	0.006
*Statins*	16,812 (65.1)	8381 (64.9)	8431 (65.3)	0.008

^a^Defined as diabetic neuropathy, retinopathy, or nephropathy

*eGFR* estimated glomerular filtration rate by Chronic Kidney Disease Epidemiology formula, *oGLD* other glucose-lowering drugs, *SGLT2i* sodium–glucose co-transporter inhibitors

In the SGLT2i group, the overall exposure to the drug class was 9484 years. The dapagliflozin, empagliflozin and canagliflozin accounted for 60%, 26%, and 14% of the total exposure time, respectively. The overall exposure time for oGLDs was 10,012 years (see Additional file 1: Table S4 for detailed information on exposure time for the different drugs). New users of dipeptidyl peptidase-4 inhibitors (DPP4i) had the highest proportion of exposure time (20.8%), followed by metformin (20.7%), insulin (20.6%), sulphonylurea (15.5%), glucagon-like peptide-1 receptor agonists (GLP1RA; 10.3%), meglitinide (9.1%), and other drugs (3.4%).

### Incidence rate and risk of CV events and renal impairment

**Fig. 1:**
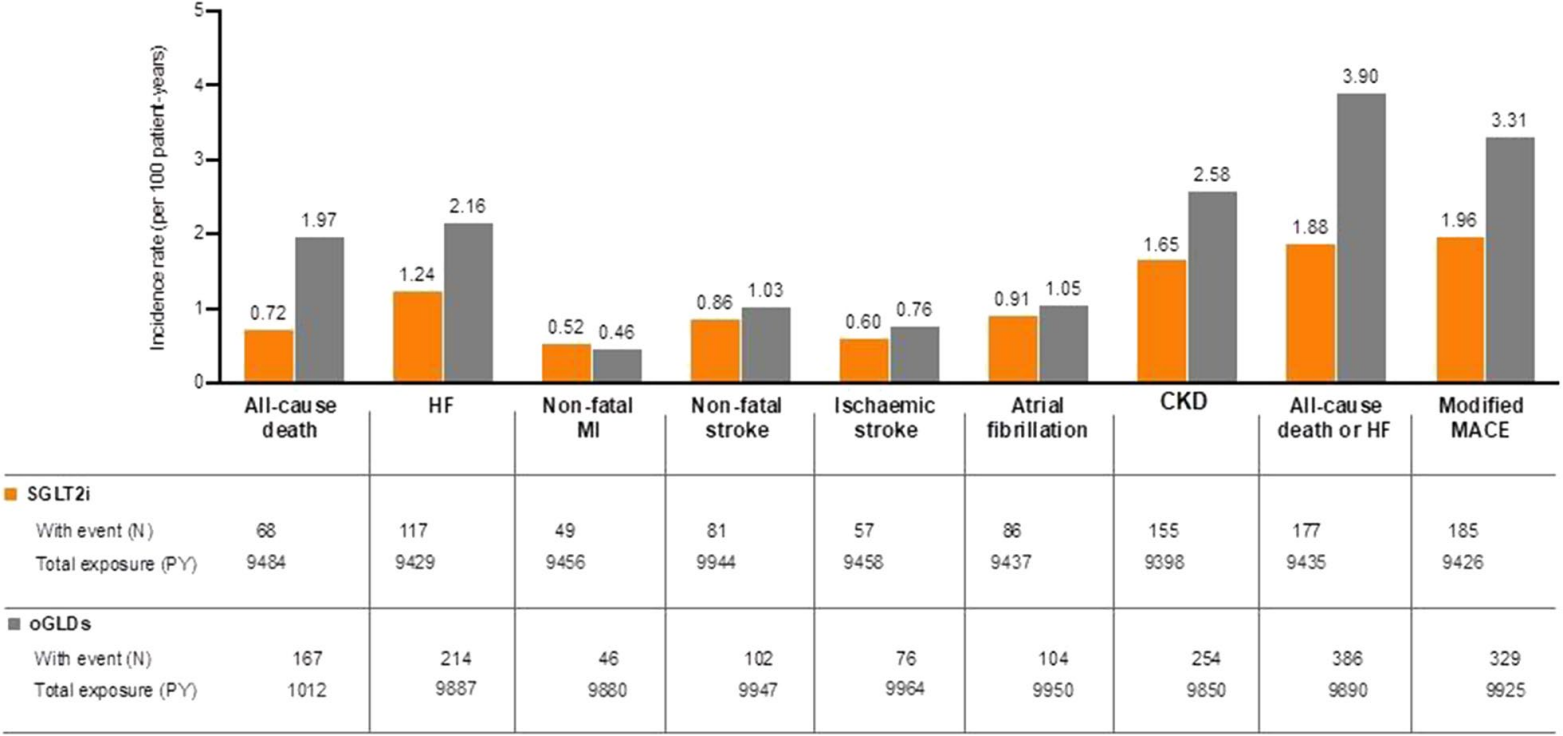
Incidence rates of the different events per 100 person-years by treatment. *CKD* chronic kidney disease, *HF* heart failure, *MACE* major adverse cardiovascular events, *MI* myocardial infarction, *oGLDs* other glucose-lowering drugs, *PY* patient-years of exposure, *SGLT2i* sodium–glucose co-transporter inhibitors

At baseline, 27% of patients had established CVD, and 6.6% CKD. The incident rate (i.e., new events per 100 patient-years) of HF, all-cause death, the combination of both outcomes, and modified MACE was lower in patients with T2DM receiving SGLT2i than in patients initiating oGLDs (IR = 1.24 vs. 2.16, 0.72 vs. 1.97, 1.88 vs. 3.90, and 1.96 vs. 3.31, respectively; Fig. 1). However, the IRs of the other studied CV events (i.e., non-fatal MI, non-fatal stroke, ischemic stroke, and atrial fibrillation) were similar between the two groups (Fig. 1). Finally, the occurrence of CKD during the follow-up period was lower after initiation of SGLT2i compared with initiation of oGLDs (IR 1.65 vs. 2.58).

**Fig. 2:**
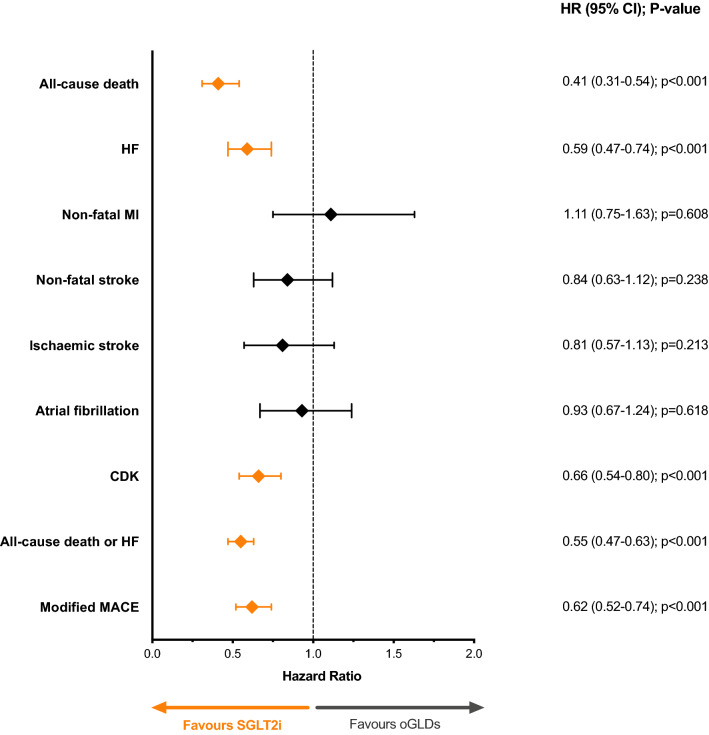
Forest plot of the adjusted incident hazard ratios (HR) and 95% CI for the different outcomes. *CKD* chronic kidney disease, *CI* confidence interval, *HF* heart failure, *MACE* major adverse cardiovascular events, *HR* hazard ratio, *MI* myocardial infarction, *oGLDs* other glucose-lowering drugs, *PY* patient-years of exposure, *SGLT2i* sodium–glucose co-transporter inhibitors

The initiation of an SGLT2i was associated with 41% lower adjusted risk of incident HF, 59% lower risk of all-cause death, 54% lower risk of the composite outcome, and 38% lower risk of modified MACE compared with matched patients on oGLDs (all p < 0.001; Fig. 2). In contrast, no statistically significant difference was detected in the risk for incident non-fatal MI, non-fatal stroke, ischemic stroke, and atrial fibrillation between the two treatment groups. Lastly, we observed that patients receiving SGLT2i had significantly reduced incident CKD compared with those on oGLDs (p < 0.001; Fig. 2).

### Sensitivity and subgroup analyses

Similar results regarding the number of events and IRs were observed for all of the study outcomes in the sensitivity analysis using the intention-to-treat approach population (Additional file 1: Table S5 and http://dapcat.shinyapps.io/CVD_REAL). Moreover, crude and adjusted Cox regression analyses (i.e., by gender, age, T2DM duration, hypertension, BMI, and antihypertensive drugs) showed magnitude and effects similar to the ones detected in the on-treatment analysis approach (Additional file 1: Table S6 and http://dapcat.shinyapps.io/CVD_REAL).

The subgroup analyses showed no statistically significant interactions with users’ characteristics, including age (≥ 65 years), gender, comorbidity profile (i.e., prior CVD, HF, or CKD), or CV and baseline medication (i.e., treatment with antihypertensive drugs, insulin, sulfonylureas, GLP1-RA thiazolidinedione, and statins). As such, none of the these variables modified the association between SGLT2i use and reduced risk of HF, all-cause death, all-cause death or HF, MACE, and CKD (Additional file 1: Figures S3 to S11 or http://dapcat.shinyapps.io/CVD_REAL).

## Discussion

In the present study involving a primary care population with T2DM from Catalonia starting new antidiabetic treatment we observed a significantly lower risk of HF associated with initiation of SGLT2i compared with matched patients initiating oGLDs. We also detected lower risk of all-cause mortality, CKD, modified MACE, and all-cause death or HF. In contrast, we did not identify any significant differences between groups regarding MI, non-fatal stroke, ischemic stroke and atrial fibrillation.

Several real-world observational studies have been published with routinely collected data from different countries [18–22, 39–41]. The baseline characteristics of the subjects included in our study are similar to those reported regarding age, prevalence of CVD, microvascular complications, and prior CV therapies. Moreover, the highest proportion of exposure time was to dapagliflozin, which was also the case in other RW studies (60% in our population, and between 51 and 94% in other observational studies) [18–22, 41].

The main outcome of our study was the reduction in the incidence rate of HF, which is a common complication associated with CV death in T2DM [5]. The relative risk reduction of HF was 41%, which is in line with the 30–39% reduction observed in other RW studies for hospitalizations for HF [18–22, 41]. In addition, the risk reduction for HF or all-cause mortality was 45% in our analysis, which is as well in agreement with the 31–46% range reported in observational studies [18–21, 40]. It is possible that the greater risk reduction observed in our study was due to the fact that we had no data on hospital events for HF admissions. Indeed, the need for hospitalization among patients with HF is frequent due to recurrent decompensation, with an estimated 12–45% incidence of hospitalizations for HF at 1 year [42, 43]. In addition, we had a higher exposition to empagliflozin in our study compared with the other observational studies (26% vs. 5–9%) [22, 27, 28]. The users who initiated SGLT2i in our study reported 68 events related to all-cause mortality compared with 197 events in the oGLD group, corresponding to a risk reduction of 59%. These results were slightly higher, but in line with the other real-world studies, in which the risk reduction of all-cause mortality ranged from 46 to 56% [18–22, 37–39]. It could be hypothesized that the differences between our results and those of other RCTs or RW studies could be related to different exposure to the individual SGLT2i across studies. However, the relative effectiveness of each product on the different outcomes remains to be elucidated, because study populations and designs are different. Recent attempts to indirectly address this issue through network meta-analyses led to differing conclusions regarding the differential effect of individual SGLT2i on outcomes such as HF and all-cause mortality [44, 45]. Therefore, in the absence of prospective or retrospective head-to-head trials comparing individual SGLT2i, it cannot be ascertained to what extent the exposure to the individual SGLT2i in CVD-REAL Catalonia vs. other studies could have contributed to the observed differences.

New use of SGLT2i was associated with a 38% lower risk of MACE compared to a new use of oGLDs, which is higher than the 21–22% reported by two other observational studies [19, 20]. In the absence of data on CV death, we used a modified MACE and replaced CV death by all-cause death. It is then conceivable that we included a greater number of events, thus resulting in a higher-than-expected reduction in this particular outcome.

In the moment of realization of the study SGLT2i were not recommended in severe renal impairment, namely < 45 mL/min/1.73 m^2^ for empagliflozin and canagliflozin, and < 60 mL/min/1.73 m^2^ for dapagliflozin [46, 47]. Regarding the assessed renal outcome, we found that the risk of CKD after initiation with SGLT2i was 34% less than in the oGLDs group, while the risk of the composite outcome eGFR decline or end stage renal disease (ESRD) was 50% lower in the CVD REAL 3 cohort [22]. On the one hand, the mean eGFR value in our study was 58.8 mL/min/1.73 m^2^, indicating a mild to moderate loss of kidney function, while in CVD REAL 3 the mean value was above 90 mL/min/1.73 m^2^ (normal range) and only 8% of initiators had values below 60 mL/min/1.73 m^2^. In that study, the annual rate of change in eGFR from baseline showed the benefit of initiating an SGLT2i regardless the eGFR subgroup, although the magnitude of the change was lower among those with compromised kidney function (30% and 79% in those with < 60 and 60–90 mL/min/1.73 m^2^, respectively). Another RW study conducted in Italy, the DARWIN-T2D, assessed the albumin excretion rate (AER) as a surrogate of kidney outcome in T2DM patients treated with dapagliflozin vs. active comparators (i.e., GLPR-1a, DPP-4i, or glicazide) [48]. The mean eGFR was 83 mL/min/1.73 m^2^ at baseline and the authors reported a decline of 37% in the AER irrespective of baseline eGFR, while there was no change among those who received a comparator. Lastly, a systematic review and meta-analysis of RCTs in patients with T2DM and CKD (defined as eGFR < 60 mL/min/1.73 m^2^) treated with SGLT2i found that these agents slowed the annual loss in kidney function (eGFR slope) and led to a 29% reduction in the risk of the composite outcome doubling serum creatinine, ESRD, or renal death [15]. A reduction of 34% vs. placebo in the composite outcome was as well reported in the CREDENCE trial, conducted in patients with T2DM and albuminuric CKD receiving canagliflozin [17]. Besides, in the DAPA-CKD trial with dapagliflozin, which included 68% of patients with CKD and T2DM, reductions of 39% and 31% vs. placebo were observed in the primary composite outcome and in all-cause mortality, respectively [16].

The incident rates of non-fatal MI, stroke (non-fatal or ischemic), and atrial fibrillation were not different between patients initiating SGLT2i or other oGLDs in our study. A neutral or small effect of SGLT2i on all of these particular outcomes has been as well observed in previous RW studies [20, 21, 39, 49]. There is also a possibility that the diagnostic codes used and proposed by the global CVD Real study protocol for these events had low prevalence in our database. Moreover, this modest or even nonsignificant association of SGLT2i with atherothrombotic events has been as well observed in several meta-analyses of RCTs comparing this drug class against placebo or active comparators [10, 50–52].

The results of our study should be considered in the context of several potential strengths and limitations. The main strength of our study is a large number of patients included the representativeness of the diabetic population [19]. Moreover, our study involves real-world data from a South European region where the prevalence of CV risk factors and CV disease in patients with T2DM is expected to be different from that in Northern Europe or the US [53]. Indeed, our results go in the same direction as other CVD-Real studies, besides differences in the studied populations. Moreover, the SIDIAP database has been extensively used for different epidemiologic and pharmacoepidemiologic national and international research, and it is established as the well-validated primary care Spanish database for the study of diabetes [33, 54]. One limitation of the study is that inherent to all observational studies based on health care records of real clinical practice, the possibility of residual and unmeasured confounding factors cannot be ruled out. However, we used robust statistical techniques, including well-balanced groups created by propensity-matching and sensitivity analyses. Another limitation is that we did not have information on CV mortality, which could have impacted the estimation of the incident rates of MACE if defined as including CV death. Moreover, our average follow-up time was relatively limited, as SGLT2i use in real-world settings is still relatively recent; thus, longer period analyses will be needed to evaluate if the positive effects of SGLT2i are sustained over time. Since the comparator group was oGLD’s in our study, there was a possibility of “immortal time bias,” which occurs when two patient groups are hierarchically formed within a time interval. However, the new user episodes design combined with propensity score matching for index date and time since initiation eliminate this bias. Furthermore, if the comparator group was DPP4i instead of OGLD´s, a similar tendency in outcomes favouring SGLT2i users was observed and published [55]. Lastly, we had limited socio- economic data and no information on lifestyle variables for patients. For instance, dietary differences between European countries may modulate CV outcomes, since it is well known that subjects highly adherent to the Mediterranean diet have about 30% lower risk of CVD morbidity and mortality, and this raises up to 40–45% in randomized clinical trials (RCTs) with patients at high CVD risk [56–58].

## Conclusion

In this real-world study of patients with T2DM attended in routine clinical practice from a South European region, new use of SGLT2i was associated with a lower risk of HF, mortality and renal events compared with the use of oGLDs. These results expand previous observational studies supporting the use of SGLT2i in patients with a broad CV risk profile in real-world clinical practice. Moreover, they show that the magnitudes of the associated CV and renal benefits are similar to those observed in other European geographical regions.

## Supplementary Information

**Additional file 1: Figure S1.** Flow chart of the patients included in the study. **Figure S2.** Pre and post propensity score matching and standardised differences. **Table S1.** Variables included in the propensity score. **Table S2.** Variables included in the adjustments of Cox regression analysis. **Table S4.** Number of patients and follow-up time (years). **Table S5.** Sensitivity crude analysis of intention-to-treat. **Table S6.** Sensitivity analysis of intention-to-treat output from Cox regression models. **Figure S3.** Subgroup analysis for heart failure outcome, including gender, age, chronic kidney disease, history of cardiovascular disease, cardiovascular and diabetes medications at baseline (ITT approach). **Figure S4.** Subgroup analysis for all-cause death or hearth failure outcome, including gender, age, chronic kidney disease, history of cardiovascular disease, cardiovascular and diabetes medications at baseline (ITT approach). **Figure S5.** Subgroup analysis for modified MACE outcome, including gender, age, chronic kidney disease, history of cardiovascular disease, cardiovascular and diabetes medications at baseline (ITT approach). **Figure S6.** Subgroup analysis for all-cause death outcome, including gender, age, chronic kidney disease, history of cardiovascular disease, cardiovascular and diabetes medications at baseline (ITT approach). **Figure S7.** Subgroup analysis for nonfatal myocardial infarction outcome, including gender, age, chronic kidney disease, history of cardiovascular disease, cardiovascular and diabetes medications at baseline (ITT approach). **Figure S8.** Subgroup analysis for non-fatal stroke outcome, including gender, age, chronic kidney disease, history of cardiovascular disease, cardiovascular and diabetes medications at baseline (ITT approach). **Figure S9.** Subgroup analysis for ischemic stroke outcome, including gender, age, chronic kidney disease, history of cardiovascular disease, cardiovascular and diabetes medications at baseline (ITT approach). **Figure S10.** Subgroup analysis for atrial fibrillation outcome, including gender, age, chronic kidney disease, history of cardiovascular disease, cardiovascular and diabetes medications at baseline (ITT approach). **Figure S11.** Subgroup analysis for chronic kidney disease outcome, including gender, age, chronic kidney disease, history of cardiovascular disease, cardiovascular and diabetes medications at baseline (ITT approach). **Table S7.** Specification of diseases codes used. **Table S8.** Specification of ATC/DDD codes used.

## Data Availability

The data controller for SIDIAP does not allow the sharing of raw data. Statistical codes are available upon request from the corresponding authors (J.F.-N or D.M).

## Abbreviations

CVD: Cardiovascular disease
CKD: Chronic kidney disease
HF: Heart failure
MACE: Major adverse cardiovascular outcomes
MI: Myocardial infarction
oGLDs: Other glucose-lowering drugs
RW: Real world
RCT: Randomized clinical trials
SGLT2i: Sodium–glucose co-transporter-2 inhibitors
T2DM: Type 2 diabetes mellitus
