# Supplementary material for: Cardiovascular and mortality benefits of sodium–glucose co-transporter-2 inhibitors in patients with type 2 diabetes mellitus: CVD-Real Catalonia
Source: Cardiovasc Diabetol. 2021 Jul 9;20:139. doi: 10.1186/s12933-021-01323-5 (PMC8272340; doi:10.1186/s12933-021-01323-5)
Supplement: Supplementary file 1 — Additional file 1: Figure S1. Flow chart of the patients included in the study. Figure S2. Pre and post propensity score matching and standardised differences. Table S1. Variables included in the propensity score. Table S2. Variables included in the adjustments of Cox regression analysis. Table S4. Number of patients and follow-up time (years). Table S5. Sensitivity crude analysis of intention-to-treat. Table S6. Sensitivity analysis of intention-to-treat output from Cox regression models. Figure S3. Subgroup analysis for heart failure outcome, including gender, age, chronic kidney disease, history of cardiovascular disease, cardiovascular and diabetes medications at baseline (ITT approach). Figure S4. Subgroup analysis for all-cause death or hearth failure outcome, including gender, age, chronic kidney disease, history of cardiovascular disease, cardiovascular and diabetes medications at baseline (ITT approach). Figure S5. Subgroup analysis for modified MACE outcome, including gender, age, chronic kidney disease, history of cardiovascular disease, cardiovascular and diabetes medications at baseline (ITT approach). Figure S6. Subgroup analysis for all-cause death outcome, including gender, age, chronic kidney disease, history of cardiovascular disease, cardiovascular and diabetes medications at baseline (ITT approach). Figure S7. Subgroup analysis for nonfatal myocardial infarction outcome, including gender, age, chronic kidney disease, history of cardiovascular disease, cardiovascular and diabetes medications at baseline (ITT approach). Figure S8. Subgroup analysis for non-fatal stroke outcome, including gender, age, chronic kidney disease, history of cardiovascular disease, cardiovascular and diabetes medications at baseline (ITT approach). Figure S9. Subgroup analysis for ischemic stroke outcome, including gender, age, chronic kidney disease, history of cardiovascular disease, cardiovascular and diabetes medications at baseline (ITT approach). Figure S10. Subgrou [file 12933_2021_1323_MOESM1_ESM.docx]

**ONLINE-Only Additional Materials**

These Additional materials have been provided by the authors to give the readers additional information about the study.

**Cardiovascular and Mortality Benefits of Sodium-glucose co-transporter-2 Inhibitors in Patients with Type 2 Diabetes Mellitus: CVD-Real Catalonia**

Jordi Real ^1,2^, Bogdan Vlacho ^1^, Emilio Ortega^3, 4,^, Joan Antoni Vallés ^1^,^5^, Manel Mata-Cases ^1,2,6^, Esmeralda Castelblanco^1,2^, Eric T. Wittbrodt ^7^, Peter Fenici ^8^, Mikhail Kosiborod ^9^, Dídac Mauricio ^1,2,10,11^ * and Josep Franch-Nadal ^1,2,12^*

**Figure S1.** Flow chart of the patients included in the study

**
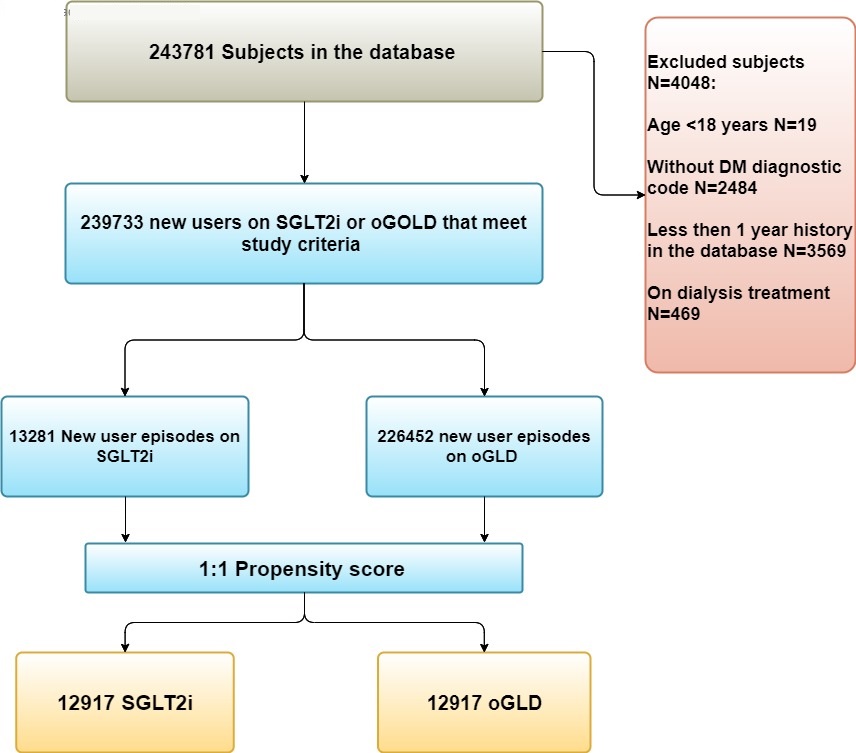
**

**Figure S2.** Pre and post propensity score matching and standardised differences

**
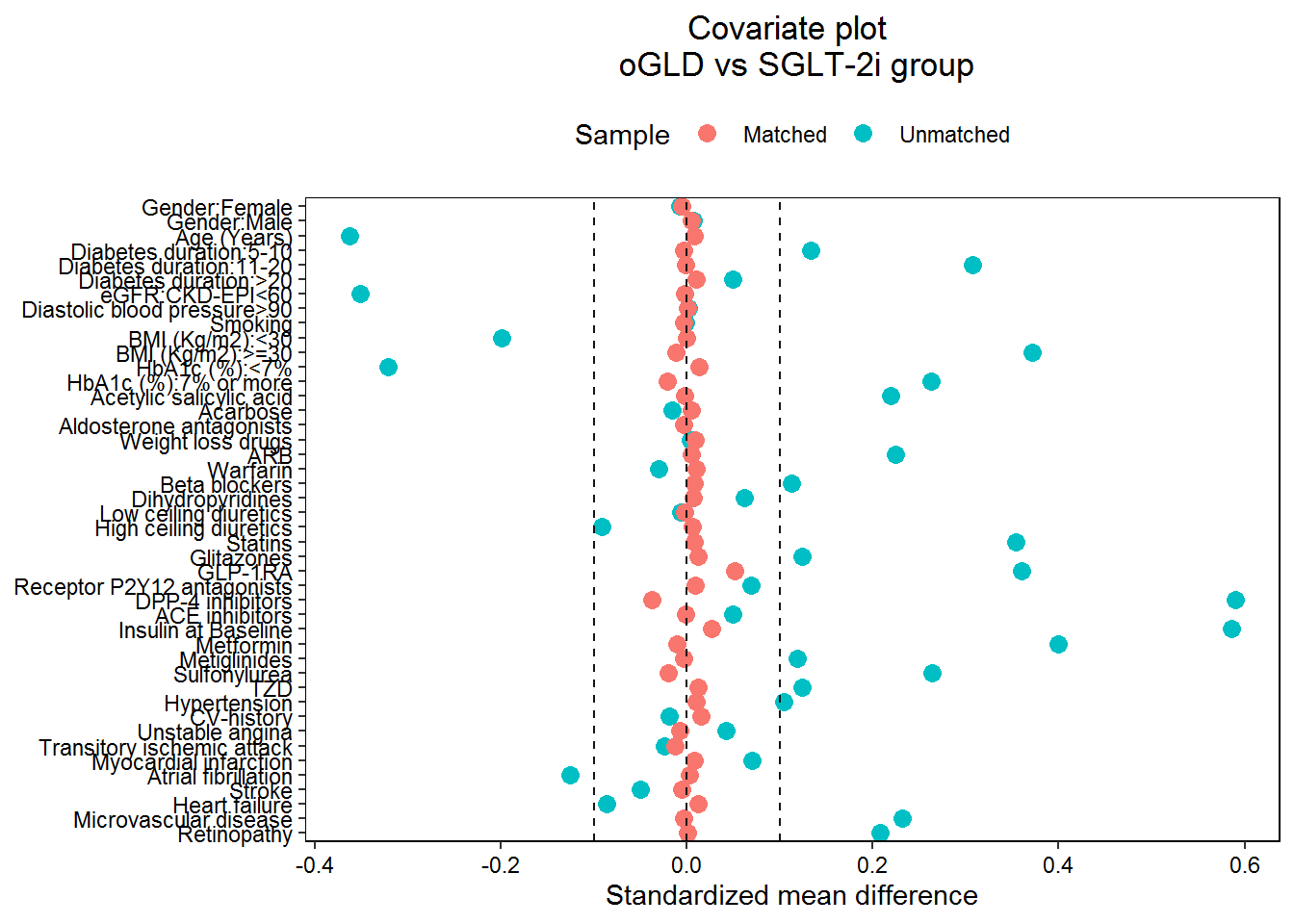
**

**Table S1.** Variables included in the propensity score

| **Propensity score variables** |
| --- |
| Gender |
| Age (Years) |
| Duration of Type 2 diabetes |
| Year-semester of Index date |
| Deprivation index) |
| estimate Glomerular Filtration Rate <60 |
| Chronic kidney disease according eGFR or CAC |
| Peripheral artery disease >90 |
| Smoking |
| Body mass index |
| HbA1c |
| Low dose acetylic salicylic acid |
| Acarbose |
| Aldosterone antagonists |
| Weight loss drugs |
| Angiotensin II receptor blockers |
| Warfarin |
| Beta blockers |
| Dihydropyridines |
| Low ceiling diuretics (thiazides) |
| High ceiling diuretics (loop-diuretics) |
| Statins |
| Glitazones |
| GLP-1RA |
| Receptor P2Y12 antagonists |
| DPP-4 inhibitors |
| Angiotensin-converting enzyme inhibitors |
| Insulin at Baseline |
| Metformin |
| Meglitinide |
| Sulfonylurea |
| Thiazolidinedione |
| Hypertension |
| Cardiovascular history |
| Unstable angina |
| Transitory ischemic attack |
| Myocardial infarction |
| Atrial fibrillation |
| Stroke |
| Heart failure |
| Microvascular disease |
| Retinopathy |

**Table S2.** Variables included in the adjustments of Cox regression analysis

| **Adjustment variables** |
| --- |
| Group |
| Gender |
| Age (Years) |
| Duration of Type 2 diabetes |
| Hipertension (PAS/PAD>140/90) |
| Body mass index |
| Beta blockers |
| Non-hydropyridines (calcium channel blockers) |
| Low ceiling diuretics (thiazides) |
| High ceiling diuretics (loop-diuretics) |
| RAAS inhibitors (ARB/ACEi) |
| Myocardial infarction |
| Heart failure |
| Angiotensin II receptor blockers |
| ACE inhibitors |

**Table S3.**

|  | **All** | **oGLD** | **SGLT2i** |
| --- | --- | --- | --- |
| N | 239,733 | 226,452 | 13,281 |
| Gender (female) | 105,653 (44.1%) | 99,845 (44.1%) | 5,808 (43.7%) |
| Age (Years) | 66.4 (13.1) | 66.6 (13.2) | 62.8 (10.4) |
| Cardiovascular disease | 67,827 (28.3%) | 64,178 (28.3%) | 3,649 (27.5%) |
| Heart failure | 17,902 (7.5%) | 17,160 (7.6%) | 742 (5.6%) |
| Myocardial infarction | 11,730 (4.9%) | 10,863 (4.8%) | 867 (6.5%) |
| Unstable angina | 2,333 (1%) | 2,140 (1.0%) | 193 (1.5%) |
| Atrial fibrillation | 18,664 (7.8%) | 17,978 (7.9%) | 686 (5.2%) |
| Stroke | 16,313 (6.8%) | 15,556 (6.9%) | 757 (5.7%) |
| Peripheral artery disease | 16,184 (6.8%) | 15,209 (6.7%) | 975 (7.3%) |
| Microvascular disease | 32,159 (13.4%) | 29,164 (12.9%) | 2,995 (22.6%) |
| Cancer | 32,910 (13.7%) | 31,487 (13.9%) | 1,423 (10.7%) |
| Deprivation index (medium-high) | 116,940 (48.8%) | 110,559 (48.9%) | 6,381 (48.1%) |
| Diabetes duration (≥5 years) | 136,799 (57%) | 126,444 (55.8%) | 10,355 (78.0%) |
| Acarbose | 357 (0.2%) | 357 (0.2%) | 0 (0.0%) |
| Insulin | 35,015 (14.6%) | 35,015 (15.5%) | 0 (0.0%) |
| GLP1RA | 6,311 (2.6%) | 6,311 (2.8%) | 0 (0.0%) |
| IDPP4 | 48,137 (20.1%) | 48,137 (21.3%) | 0 (0.0%) |
| SGTL2i | 13,281 (5.5%) | 0 (0.0%) | 13,281 (100%) |
| Metformin | 84,862 (35.4%) | 84,862 (37.5%) | 0 (0.0%) |
| Meglitinide | 14,926 (6.2%) | 14,926 (6.6%) | 0 (0.0%) |
| Sulphonylurea | 34,785 (14.5%) | 34,785 (15.4%) | 0 (0.0%) |
| Thiazolidinedione | 2,059 (0.9%) | 2,059 (0.9%) | 0 (0.0%) |
| eGFR | 56.9 (8.00) | 56.7 (8.16) | 59.0 (3.95) |
| CKD-EPI<60 | 36,761 (15.3%) | 35,842 (15.8%) | 919 (6.9%) |
| CKD according eGFR or CAC | 54,528 (22.7%) | 52,030 (23.0%) | 2,498 (18.8%) |
| Hipertension (PAS/PAD>140/90) | 61,689 (25.7%) | 58,064 (25.6%) | 3,625 (27.3%) |
| Smoking | 38,267 (16.0%) | 36,155 (16.0%) | 2,112 (15.9%) |
| Body mass index | 31.1 (5.67) | 31.0 (5.64) | 33.3 (5.74) |
| HbA1c (%) | 8.26 (1.69) | 8.24 (1.70) | 8.61 (1.48) |
| Antihypertensive | 160,229 (66.8%) | 150,110 (66.3%) | 10,119 (76.2%) |
| ARB | 56,688 (23.6%) | 52,217 (23.1%) | 4,471 (33.7%) |
| Beta blockers | 53,733 (22.4%) | 50,130 (22.1%) | 3,603 (27.1%) |
| Calcium channel blockers | 5,574 (2.33%) | 5,319 (2.4%) | 255 (1.92%) |
| Low ceiling diuretics (thiazides) | 22,918 (9.6%) | 21,673 (9.6%) | 1,245 (9.4%) |
| High ceiling diuretics (loop-diuretics) | 34,773 (14.5%) | 33,216 (14.7%) | 1,557 (11.7%) |
| ACE inhibitors | 86,409 (36.0%) | 81,323 (35.9%) | 5,086 (38.3%) |
| Statins | 119,424 (49.8%) | 110,702 (48.9%) | 8,722 (65.7%) |
| Hypertension | 159,857 (66.7%) | 150,410 (66.4%) | 9,447 (71.1%) |
| Year of Index date: |  |  |  |
| 2013 | 6,224 (2.6%) | 6,198 (2.74%) | 26 (0.2%) |
| 2014 | 75,885 (31.7%) | 75,097 (33.2%) | 788 (5.9%) |
| 2015 | 77,459 (32.3%) | 72,884 (32.2%) | 4,575 (34.4%) |
| 2016 | 80,165 (33.4%) | 72,273 (31.9%) | 7,892 (59.4%) |
| Days of follow-up | 524 (330) | 536 (332) | 331 (234) |

**Table S4.** Number of patients and follow-up time (years)

| **Drugs** | **N** | **Sum** | **Mean** | **SD** | **Max** |
| --- | --- | --- | --- | --- | --- |
| SGTL2i | 12,917 | 9,483.52 | 0.73 | 0.57 | 3.06 |
| Dapagliflozin | 6,692 | 5,686.18 | 0.85 | 0.65 | 3.06 |
| Canagliflozin | 2,062 | 1,333.79 | 0.65 | 0.46 | 1.66 |
| Empagliflozin | 4,163 | 2,463.55 | 0.59 | 0.43 | 1.72 |
|  |  |  |  |  |  |
| oGLD | 12,917 | 10,012.01 | 0.78 | 0.62 | 3.07 |
| Acarbose | 30 | 19.48 | 0.65 | 0.53 | 2.80 |
| Insulin | 2,466 | 2,069.92 | 0.84 | 0.66 | 3.07 |
| GLP1RA | 1,321 | 1,035.32 | 0.78 | 0.59 | 2.98 |
| IDPP4 | 2,804 | 2,083.04 | 0.74 | 0.58 | 2.96 |
| Metformin | 2,679 | 2,081.39 | 0.78 | 0.63 | 3.04 |
| Meglitinide | 1,279 | 910.83 | 0.71 | 0.60 | 3.05 |
| Sulphonylurea | 2,026 | 1,561.32 | 0.77 | 0.62 | 3.07 |
| Thiazolidinedione | 312 | 250.70 | 0.80 | 0.67 | 3.04 |

**Table S5.** Sensitivity crude analysis of intention-to-treat

| **Grup** | **Event** | **PYear** | **Events** | **Rate/100 pyear** |
| --- | --- | --- | --- | --- |
| oGLD | Heart failure | 11,745.96 | 244 | 2.08 |
| SGLT2 | Heart failure | 11,659.24 | 164 | 1.41 |
| oGLD | All-casuse death or heart failure | 11,745.96 | 485 | 4.13 |
| SGLT2 | All-casuse death or heart failure | 11,659.24 | 263 | 2.26 |
| oGLD | modified MACE | 11,789.12 | 432 | 3.66 |
| SGLT2 | modified MACE | 11,662.54 | 255 | 2.19 |
| oGLD | All-cause death | 11,922.32 | 270 | 2 .26 |
| SGLT2 | All-cause death | 11,763.23 | 112 | 0.95 |
| oGLD | Myocardial infarction | 11,879.50 | 54 | 0.45 |
| SGLT2 | Myocardial infarction | 11,724.25 | 56 | 0.48 |
| oGLD | Stroke | 11,830.85 | 123 | 1.04 |
| SGLT2 | Stroke | 11,699.72 | 96 | 0.82 |
| oGLD | Ischemic stroke | 11,850.74 | 94 | 0.79 |
| SGLT2 | Ischemic stroke | 11,715.42 | 74 | 0.63 |
| oGLD | Atrial fibrillation | 11,833.81 | 119 | 1.01 |
| SGLT2 | Atrial fibrillation | 11,689.60 | 105 | 0.90 |
| oGLD | Kidney disease | 11,703.00 | 300 | 2.56 |
| SGLT2 | Kidney disease | 11,599.45 | 212 | 1.83 |

**Table S6**. Sensitivity analysis of intention-to-treat output from Cox regression models

|  | **PT.Year** | **EVENTS** | **HR** | **IC951** | **IC952** | **p** |
| --- | --- | --- | --- | --- | --- | --- |
| All-cause death ITT | 23,685.55 | 382 | 0.423 | 0.341 | 0.524 | 0.000 |
| ITT adjusted | 23,685.55 | 382 | 0.457 | 0.368 | 0.569 | 0.000 |
| Heart failure ITT | 23,405.20 | 408 | 0.672 | 0.553 | 0.816 | 0.000 |
| ITT adjusted1 | 23,405.20 | 408 | 0.700 | 0.575 | 0.852 | 0.000 |
| Atrial fibrillation ITT | 23,523.41 | 224 | 0.890 | 0.693 | 1.142 | 0.359 |
| ITT adjusted2 | 23,523.41 | 224 | 0.927 | 0.720 | 1.194 | 0.559 |
| Stroke ITT | 23,530.56 | 219 | 0.785 | 0.604 | 1.020 | 0.070 |
| ITT adjusted3 | 23,530.56 | 219 | 0.799 | 0.615 | 1.039 | 0.094 |
| Myocardial infarction ITT | 23,603.74 | 110 | 1.037 | 0.723 | 1.487 | 0.842 |
| ITT adjusted4 | 23,603.74 | 110 | 1.057 | 0.737 | 1.515 | 0.765 |
| All-casuse death or HHF | 23,405.20 | 748 | 0.546 | 0.471 | 0.632 | 0.000 |
| ITT adjusted | 23,405.20 | 748 | 0.577 | 0.497 | 0.670 | 0.000 |
| Ischemic stroke | 23,566.17 | 168 | 0.793 | 0.588 | 1.067 | 0.126 |
| ITT adjusted1 | 23,566.17 | 168 | 0.808 | 0.601 | 1.086 | 0.158 |
| Modified MACE | 23,451.65 | 687 | 0.596 | 0.513 | 0.693 | 0.000 |
| ITT adjusted2 | 23,451.65 | 687 | 0.620 | 0.533 | 0.721 | 0.000 |
| Kidney disease | 23,302.45 | 512 | 0.714 | 0.599 | 0.851 | 0.000 |
| ITT adjusted3 | 23,302.45 | 512 | 0.730 | 0.612 | 0.871 | 0.000 |

**Figure S3**. Subgroup analysis for heart failure outcome, including gender, age, chronic kidney disease, history of cardiovascular disease, cardiovascular and diabetes medications at baseline (ITT approach)

**Figure S4.** Subgroup analysis for all-cause death or hearth failure outcome, including gender, age, chronic kidney disease, history of cardiovascular disease, cardiovascular and diabetes medications at baseline (ITT approach)

**Figure S5.** Subgroup analysis for modified MACE outcome, including gender, age, chronic kidney disease, history of cardiovascular disease, cardiovascular and diabetes medications at baseline (ITT approach)

**Figure S6.** Subgroup analysis for all-cause death outcome, including gender, age, chronic kidney disease, history of cardiovascular disease, cardiovascular and diabetes medications at baseline (ITT approach)

**Figure S7.** Subgroup analysis for nonfatal myocardial infarction outcome, including gender, age, chronic kidney disease, history of cardiovascular disease, cardiovascular and diabetes medications at baseline (ITT approach)

**Figure S8.** Subgroup analysis for non-fatal stroke outcome, including gender, age, chronic kidney disease, history of cardiovascular disease, cardiovascular and diabetes medications at baseline (ITT approach)

**Figure S9.** Subgroup analysis for ischemic stroke outcome, including gender, age, chronic kidney disease, history of cardiovascular disease, cardiovascular and diabetes medications at baseline (ITT approach)

**Figure S10.** Subgroup analysis for atrial fibrillation outcome, including gender, age, chronic kidney disease, history of cardiovascular disease, cardiovascular and diabetes medications at baseline (ITT approach)

**Figure S11.** Subgroup analysis for chronic kidney disease outcome, including gender, age, chronic kidney disease, history of cardiovascular disease, cardiovascular and diabetes medications at baseline (ITT approach)

**Table S7.** Specification of diseases codes used

| **Disease** | **ICD-9** | **ICD 10** |
| --- | --- | --- |
| CVD |  |  |
| Myocardial infarction | 410 | I21-I22 |
| CABG | 414.02-07, V45.81-82 |  |
| PCI with stent |  |  |
| Unstable angina | 411 | I20.0 |
| Angina pectoris | 413, 414.0 | I20.1, I20.8, I20.9 |
| Heart failure | 428 | I50 |
| Atrial fibrillation | 427.3 | I48 |
| Stroke | 430-438, V125 | I60-I66, G45 |
| Hemorrhagic | 430-432 | I60-I62 |
| Ischemic | 433-434, 436 | I63-I64 |
| Transitory ischemic attack | V12.5, 435 | G45 |
| Peripheral artery disease | 440/441/444 | I70-I79 |
| Major organ specific bleeding | 578.0/578.1 | D629, I60, I61, I62, I850, K226, K250, K252, K254, K256, K260, K262, K264, K266, K270, K272, K274, K276, K280, K282, K284, K286, K290, K625, K920, K921, K922 |
| Bariatric surgery |  |  |
| Chronic kidney disease | 585 | N18 N08.3, E11.2, E14.2 |
| Dialysis |  | Z49 |
| Microvascular complications |  |  |
| Diabetic mono-/polyneuropathy | 354.0-355.9, 357.2, 250F | G99.0, G59.0, G63.2, E10.4, E11.4, E12.4, E13.4, E14.4 |
| Diabetic eye complications | 362, 366.41, 365.44, 362.07, 250E | H28.0, H35.8, H36.0, E10.3, E11.3, E12.3, E13.3, E14.3 |
| Diabetic foot/Peripheral angiopathy | 713.5, 250.7, 250G | E11.6B, M14.2, M14.6, M90.8, L98.4, E10.5, E11.5, E12.5, E13.5, E14.5 |
| Diabetic kidney disease | 583.81, 250D | N08.3, E10.2, E11.2, E12.2, E13.2, E14.2 |
| Diabetes with several-/unspecified complications | 250H-X | E11.6, E10.6, E13.6, E14.6, E10.7, E11.7, E12.7, E13.7, E14.7, E10.8, E11.8, E12.0, E12.8, E13.8, E14.8 |
| Severe hypoglycemia | 251,9623 | E10.0, E11.0, E12.0, E13.0, E14.0, E11.6A, E16.0-2 |
| Keto-/lactate acidosis | 249.1/250. | E10.1, E11.1, E12.1, E13.1, E14.1, E87.2 |
| Cancer | 140-239 | C00-C99 |
| COPD | 491 | J44 |
| Lower limb amputations |  |  |

CABG, coronary artery bypass grafting; PCI Percutaneous coronary intervention; CVD Cardiovascular disease, COPD: Chronic Obstructive Pulmonary Disease

**Table S8.** Specification of ATC/DDD codes used

| **Drug** | **ATC code** |
| --- | --- |
| Blood glucose lowering drugs |  |
| Metformin | A10BA |
| Sulfonylureas | A10BB |
| DPP-4 inhibitors | A10BH |
| SGLT-2 inhibitors | A10BX09, A10BX11, A10BX12 or A10BD15, A10BD16, A10BD20 |
| GLP-1RA | A10BX04, A10BX07, A10BX10, A10BX14 |
| Metiglinides | A10BX02 and A10BX03 |
| Glitazones | A10BG |
| Acarbose | A10BF |
| Insulins | A10 |
| Short-acting | A10AB |
| Intermediate-acting (isophane) | A10AC |
| Premixed insulin | A10AD |
| Long-acting | A10AE |
| Combination metformin+DPP-4 inhibitors | A10BD07, A10BD08, A10BD10 |
| Combination metformin+metiglinid | A10BD03, A10BD04, A10BD05 |
| Combination metformin+SGLT-2 inhibitors | A10BD15, A10BD16, A10BD20 |
|  |  |
|  |  |
| CVD risk treatment |  |
| Low dose acetylic salicylic acid | B01AC06 |
| Statins | C10AA |
| Antihypertensives |  |
| ACE inhibitors | C09A, C09B |
| ARB | C09C, C09D (exclude C09DX04) |
| Dihydropyridines (calcium channel blockers) | C08C |
| Low ceiling diuretics (thiazides) | C03A |
| Beta blockers | C07 |
| Non-hydropyridines (calcium channel blockers) | C08D |
| High ceiling diuretics (loop-diuretics) | C03C |
| Aldosterone antagonists | C03DA |
| Warfarin | B01AA03 |
| Receptor P2Y12 antagonists | B01AC04, B01AC22, B01AC24 |
| Other antiplatelets | B01AC07, B01AC09, B01AC11,  B01AC13, B01AC16, B01AC17, B01AC21 |
| Corticosteroids | H02 |
| Weight loss drugs | A08A |
